# The Hospital. Nursing Section

**Published:** 1902-03-01

**Authors:** 


### The Hospital.

### Nursing Section.

Contributions for this Section of "The Hospital" should be addressed to the Editor, "The Hospital" Nursing Section, 28 & 29 Southampton Street, Strand, London, W.C.

No. 805.—Vol. XXXI. *SATURDAY, MARCH* 1, 1902.

### Notes on News from the Nursing World.

### ROYAL NATIONAL PENSION FUND FOR NURSES.

The annual general meeting will be held about the middle of April, when the chair will be taken by Mr. E. A. Hambro, chairman of the council; the exact date will be published later. Any policy-holder of the Fund who wishes to be present will be welcomed by the council.

### PRINCESS CHRISTIAN'S TRAINED NURSES.

Notwithstanding the bereavements and anxiety which the Royal President of the Princess Christian Trained Nurses' Association sustained last year, the fifteenth annual report states that Her Royal Highness paid frequent visits to the home at Windsor, and manifested the same interest as usual in the nurses and their work. The figures of the report are interesting. The number of cases nursed was 1,328, and that of visits paid 27,818. In addition to these 292 cases were privately nursed, 55 being tended at reduced fees. The permanent staff has not been increased, but four extra nurses were temporarily employed. At the annual meeting of the Egham branch of the institution, Colonel Boyes, the chairman, said that the value of really good skilful nursing had been very forcibly brought home to his mind during the twelve months, for he had had two nurses in his own house for over six weeks, both of whom had come from parent institutions at Windsor. He was able to speak, from personal experience, "in the very warmest terms of the ever cheerful and untiring devotion with which they performed their arduous duties."

### THE QUEEN'S NURSES.

One of the last things said by a patient in one of the London districts, just before her death, showed her appreciation of the visits paid to her by the district nurse who was also a Queen's nurse. "We speak of our dear Queen Victoria as dead," she said, "but she can never really die as long as her nurses are going about doing for others what you do for me."

### THE WAR NURSES.

The s.s. *Lake Erie* left Capetown for England on Feb. 12th with Nursing Sisters M. E. Loveday and A. J. Garlich on board; and the s.s. *Orient* on Feb. 13th, with Nursing Sister S. E. Webb. The s.s. *Nubia*, which quitted Port Natal on the 20th, has on board Nursing Sisters M. Steenson, J. H. Roberts, J. E. Scott, M. T. Neville, E. J. Martin, E. A. Cox, H. P. N. Ferreira, L. G. Keel, L. C. M. Noble, and E. Prangby.

### OFFICERS AND "SIEGE NURSES."

Soon after the relief of Ladysmith, Mrs. Ludlow, of the Army Nursing Service Reserve, late lady superintendent of the Royal Free Hospital, Gray's Inn Road, received a letter stating that the officers of the Cavalry Brigade and the Imperial Light Horse stationed in Ladysmith during the siege wished to send to all of the "Siege Nurses" a small token of their appreciation of the care and devotion which had been shown to their wounded men. Mrs. Ludlow, who is now in Ireland, has just had forwarded to her address a gold brooch with a red enamelled Maltese cross in the centre, and in the centre of the cross a diamond star, with "Ladysmith, 1900" beneath the cross, her name being inscribed on the reverse. This graceful mode of recognising services bravely rendered will, we are sure, afford pleasure to all the recipients.

### MEDALS FOR ARMY NURSES.

It appears that there are still some people who are dissatisfied with the manner in which medals are given, or withheld, from nurses who have served in South Africa during the war. In the House of Commons last Thursday Mr. Claude Lowther asked "whether it was true that women employed in the Army Nursing Service in South Africa were not given a medal unless they held a three years' hospital certificate?" Mr. Brodrick in reply said: "The South African war medal is given to all nurses and nursing sisters who served in South Africa from October 11, 1899, but the term 'nurses and nursing sisters' is held to apply only to those specially appointed as such, and to the staff of any local charitable institution in South Africa duly recognised by the military authorities there and utilised by them for the reception and care of the sick and wounded." It can scarcely be a grievance that medals are not bestowed upon nurses who are not recognised by the military authorities.

### NURSING AT THE CONCENTRATION CAMPS.

The Government, it may confidently be assumed, will act on the recommendation of the committee of ladies who have just issued their report on the Concentration Camps in South Africa, and increase the number of qualified nurses. Indeed, to some extent the recommendation has been anticipated; for, as our readers know, a very considerable number of nurses have lately been dispatched to South Africa under the auspices of the Colonial Office. One of the colleagues of Mrs. Fawcett was a highly-trained nurse, and we have no doubt that this lady, Miss Brereton, proved herself a valuable member of the committee. It is satisfactory to find that the committee have nothing but praise for the nursing staff as at present constituted. The difficulties of the nurses must, however, be many and great. Thus, we read in the report that one woman whose children were ill with measles, painted their bodies with common green oil paint, and in the case of one added a plaster made of American leather thickly daubed with the same paint. Both the children died of arsenical poisoning. Another woman, in a far distant camp, visiting in hospital her child, who had herpes round the mouth, seized the opportunity when the nurse's back was turned to paint its face and lips with vermilion oil paint. Fortunately, this was discovered while the paint was still wet and could be easily removed. A third, whose child was seriously ill with double pneumonia, varnished the whole of its chest and stomach. In one camp a newly-confined woman was found by the camp matron drinking a mixture of brick dust and brandy. A camp nurse told the committee that, in one instance, she had put a linseed poultice on a pneumonia patient, and returning presently to see how she was progressing, it was discovered that the woman had eaten the poultice. These citations are sufficient to afford nurses who may propose to offer themselves for service in the Concentration Camps, some idea of the extraordinary vigilance required from them—a kind of vigilance of which they can have had little experience in an English hospital.

### NO EPIDEMIC AT THE LONDON HOSPITAL.

It was stated the other day that "no fewer than sixty of the nurses at the London Hospital were suffering from influenza." The statement is a ridiculous exaggeration. We are authoritatively informed that there has been nothing approaching an influenza epidemic among the nursing staff at the London. The fact is that as usual at this season of the year a large number of nurses have been off duty with a variety of minor ailments such as coughs and colds. On the other hand, although influenza has not made its ravages seriously felt amongst the nurses, the members of the private staff at the London have been extremely busy attending influenza and other patients. The wards also have been very full.

### REPORT OF THE NURSES' CO-OPERATION.

So far as the receipts of the Nurses' Co-operation during the year 1901 are concerned, there is no cause for concern. According to the report of the Finance Committee the gross receipts from patients amount to £45,014, an increase of £1,317 on the previous year. Of this sum it is stated £41,629 has been paid to nurses, an increase of £1,213. The increase of the society, derived from commission on fees, was £3,384, an increase of £102. In future, it is announced, nurses who joined the co-operation during the first five years of its existence are to be charged 5 per cent., instead of 7½ per cent., on the fees they take. But it would have been more prudent to have deferred this concession until the Howard de Walden Home can be made to pay its expenses. Last year there was a deficit of £251. As the receipts were £1,458, the expenses should have been covered, and the inference is that there has not been sufficiently close attention to details. Turning to the statement of the committee of management we find it is mentioned that they have determined not to increase the staff beyond 500 members. As there are now 492 nurses on the general staff, as well as 20 asylum-trained nurses who take mental cases only, the limit is nearly reached. Allusion is made to the right of the nurses to elect 8 of the 14 members of the committee of management, and the report says, "The committee realise the difficulty which private nurses have in attending meetings regularly, and they have under consideration a plan for securing to them a larger representation, which may bring more of them into the direct administration of the business of the Co-operation." The sooner this is done the better.

### THE SEVEN NURSES AT MILE END INFIRMARY.

Dr. Harley Brooks, medical superintendent at Mile End Infirmary, sends us a letter in defence of the non-vaccination or non-revaccination of the seven nurses who caught small-pox. We regret that our communication, addressed a fortnight ago to the official representative of the Mile End Board of Guardians, did not reach the hands of Dr. Brooks, as we are quite sure that if it had done so he would have answered it without delay. With regard to the details which Dr. Brooks controverts, the statement that none of the seven nurses had been vaccinated appeared in the *Times*; and though it seems that "each had been vaccinated in childhood," Dr. Brooks admits that the seven who contracted the disease had either not been revaccinated, or had only been revaccinated during the incubation period. The fact is that when Dr. Brooks says he agrees with us that "revaccination is a proper precaution to be taken by all nurses," he concedes, so far as we are concerned, the whole point at issue. We neither accused him nor any other particular person of disgraceful neglect, but we maintain that when small-pox made its reappearance in London the authorities of the Mile End Union should have insisted, as other authorities in London have insisted, upon every nurse in their employ being revaccinated on pain of instant dismissal. This was the only safe course to pursue, and those authorities who did not pursue it expose themselves to the charge of disgraceful neglect, though individual officials may plausibly plead that the system which allows nurses to please themselves at a time of public peril is at fault.

### THE NEW SUPERINTENDENT AT STEYNING UNION INFIRMARY.

The Guardians of the Steyning Union are to be congratulated upon their appointment of the superintendent nurse to the Infirmary. Miss Ada Clarke has had just the kind of experience which should admirably qualify her for her new position. In addition to her valuable general training at Birmingham Infirmary, where the matron found her unusually capable and intelligent, she has had the advantage of fever training, and she also possesses the L.O.S. certificate. Since October 1899 she has been connected with the Union Hospital at Stockport, first as home sister and night superintendent, and afterwards as assistant matron. Of her work in that institution the medical superintendent and the matron speak in the very highest terms; and while both are sorry to lose her services, they are satisfied that the loss of Stockport will be the gain of Steyning.

### OPENING OF A NURSES' HOME AT WOOLWICH.

A new home for the nursing staff attached to the Woolwich Workhouse Infirmary was opened last week. It stands at the rear of the infirmary in High Street, Plumpstead, and contains 45 bedrooms in addition to day and recreation rooms. The cost of the building was £10,140, another £450 having been spent in furnishing. The Guardians were fortunately able to reduce the cost of the undertaking by utilising their own freehold land for a site. Colonel Hughes, M.P., accompanied by his wife, performed the formal ceremony, and handed the key of the Home to Canon Escreet, the Chairman of the Board of Guardians.

### DEARTH OF NURSES AT GUERNSEY.

We hear that there is a want of trained nurses in Guernsey. The staff at the Victoria Cottage Hospital, Amherst, is small, and can, apparently, give no outside help or at any rate not sufficient. The friends of a young lady who was lately taken seriously ill were compelled, accordingly, to remove her to one of the hospitals. The removal may be for the advantage of the patient, but this does not alter the fact that the supply of nurses runs short in the island. Perhaps its reputation for healthiness is the cause of the scarcity.

### FRAUD AND CARELESSNESS.

After the experience of Father Raleigh, of St. Monica's Priory, Hoxton, we should like to think that the mere fact of a woman being attired in the garb of a hospital nurse, and being able to tell a plausible story of thrilling adventures, will not suffice to induce any ordinarily cautious person to assist her in her alleged need. The woman, in this instance, who wore hospital uniform in the dock where she appeared to answer the charge of obtaining £1 by false pretences, stated that she had done duty in the service hospitals in South Africa, and that she was at present engaged at Charing Cross Hospital. The sovereign which the priest gave her was obtained on the plea that she wanted clothing for herself. But, even if it did not occur to him that a nurse on the staff at Charing Cross Hospital could not be reduced to the necessity of begging for money to buy clothing, he might have taken the simple precaution to make inquiries at that institution before he parted with his coin. The sentence of 12 months' imprisonment, with hard labour, which was very fitly imposed upon the swindler, was due to evidence that she had been living a life of fraud upon charitable persons. But, really, in the specific case of Father Raleigh, the fraud should have been impossible.

### ENNISKILLEN GUARDIANS BROUGHT TO BOOK.

The attempt of the Enniskillen Board of Guardians to retain an admittedly unqualified person as night nurse in the workhouse infirmary, has been abandoned because the Irish Local Government refused to sanction her employment either permanently or temporarily. In fact the Board surcharged the guardians with the amount which they had paid her. This being so, the guardians, with much reluctance, and complaints about the arbitrary action of the Local Government Board, gave way, and decided to make a proper appointment. The standard of nursing is a good deal lower in some of the Irish workhouse infirmaries than it is even in similar institutions in England ; but the course pursued in respect to the Enniskillen guardians is another indication that the authorities on the other side of the Channel intend to be obeyed.

### GATESHEAD NURSES AND WORKMEN.

We regret to learn that the Gateshead Nursing Association receives very slender support from the workmen employed in the town. At the annual meeting it was mentioned that nearly 20,000 visits had been paid by the nurses during last year, an increase of more than 2,000 on the previous twelve months. Many of the cases were of a serious nature, and both required and received more than ordinary attention. Yet the income of the Association was £46 less than in 1900. This shows that, although there are plenty of people in Gateshead glad to enjoy the ministrations of the district nurses, there are not nearly enough prepared to manifest their appreciation in a practical manner. One of the speakers at the meeting generously announced his readiness to double his own and his wife's subscriptions, but it is from those who do not contribute at all that the Association have a right to expect support. A weekly trifle from each of the workmen earning full wages would make all the difference to the state of the Association's finances.

### MUNICIPAL BODIES AND NURSING.

According to the annual report of the Hammersmith and Fulham District Nursing Association, its financial position is far from satisfactory. Twice during the year the committee have had to draw on the emergency fund, and on neither occasion for anything more than the ordinary working expenses of the home. The population of the two boroughs covered by the nurses' work is over 200,000. Last year 1,097 cases were nursed, and 23,095 visits paid. The sum of £700, which is required for expenses, works out at rather less than one penny per head. The employers of labour have been approached almost entirely without success, though the working classes support the association well according to their means, and the patients make what payment they can. At the annual meeting on Wednesday last week, the Bishop of Kensington urged the claims of the association on the municipality. He said that people had come to appreciate enormously the great gift London had received in the new municipalities, and they were, he thought, solemnly bound to take an interest in everything which affected their welfare. "Among the things most nearly affecting their welfare must be placed the work of the district nurses, who had a wonderful way of rising above difficulties, and who put Christianity before the people in a beautiful and practical way."

### SHORT ITEMS.

At a special meeting of the Walker-on-Tyne Nursing Association, last week, the announcement was made that Mr. and Mrs. Henry F. Swan had offered to build a nurses' home for the association. The offer was gratefully accepted, and a sub-committee was appointed to carry out the wishes of the donors.—A very successful bazaar was held in connection with the Cleator and Cleator Moor District Nursing Association last week in the Market Hall, Cleator Moor. The total receipts for the two days exceeded £400. Of this amount the stall presided over by Mrs. Ainsworth, the President of the Association, realised £113, whilst the "Nurses'" stall, presided over by Nurses McMillan and Robinson (Cleator) and Nurse Longford (Millom), yielded the handsome sum of £103.—The next examination under the auspices of the Incorporated Society of Trained Masseuses will be held on Tuesday and Wednesday, March 18th and 19th. No application can be received after March 4th.—A medical and surgical home has been opened in Manchester under the charge of Miss Bland, who acts as Lady Superintendent. Miss Bland was formerly matron of the Manchester Royal Eye Hospital, and has also been matron of Beverley Cottage Hospital and Hull Sanatorium.

### Lectures on Gynæcology for Nurses.

BY ROBERT JARDINE, M.D., M.R.C.S., F.F.P. and S.G., F.R.S.E., Senior Physician to the Glasgow Maternity Hospital Examiner in Midwifery to the University of Glasgow.

LECTURE VI.—DISORDERS OF MENSTRUATION.

*Menorrhagia.*—By this term is meant profuse menstruation. The amount of discharge is greater in quantity and the period usually lasts longer than normal. It must be remembered that what might be a normal period for one woman would be profuse for another. Each case must, therefore, be judged by the normal periods of the patient.

*Causes.*—The condition may arise from constitutional as well as local causes. In young women the cause is frequently constitutional, while in older women it is generally local. It may occur at the beginning of menstrual life and is very common just before the menopause. A hot climate often causes it, and change from one climate to another. Malaria is another cause. A full habit of body is apt to give rise to it and it sometimes occurs with prolonged lactation. In the course of most fevers, and in cardiac, kidney, and liver diseases it is apt to occur, also in purpura hæmorrhagica, anæmia, and constipation. Local affections in which it occurs: All pelvic inflammations, fibroid tumours of the uterus, uterine polypi, hæmatocele, malignant disease of the uterus, foreign body in the uterus, or a retained piece of placenta or membranes after a full-time labour or an abortion, subinvolution, endometritis, inversion of the uterus, most displacements of the uterus, chronic ovaritis, cystic ovaries, and laceration of the cervix.

The indications of the condition are general and local. The general indications are anæmia from the loss of blood, emaciation, especially with malignant disease, hysteria, sterility, and often dyspepsia, with a feeling of languor and gnawing at the stomach.

*Local Indications.*—The period is profuse and prolonged judged by the patient's normal experience. Large clots are frequently passed. In some cases there may be no pain, while in others, it may be severe, depending upon the cause.

*Diagnosis.*—This is easily made from the prolonged and profuse discharge, but the diagnosis of the cause is the important point, and this may be very difficult. If the patient is a married woman, or an aged single woman, a local examination should always be made. In a young unmarried woman a local examination may be necessary, but in many, constitutional disease will be the cause, so it must be looked for. Of course, in all cases constitutional disease must be sought for, but especially in young patients; heart disease must never be overlooked. If the woman has recently had a child or an abortion, the chances are that the cause will be found in the uterus.

*Prognosis.*—The gravity of the condition will depend upon the cause; for instance, if malignant disease is found, the condition is exceedingly grave.

*Treatment.*—The patient must be kept at rest in bed. The bowels should be kept free. Ergot alone, or combined with quinine and iron, is often given, but one must seek for the cause, and remove it if possible. If it is a case of endometritis, or something retained, curetting, and perhaps plugging, will be necessary. If there is a uterine polypus it must be removed. Again, if the cause lies in the ovaries, they may require to be removed. In heart disease, cardiac tonics may be wanted. If the patient is very anæmic her head should be kept low, and it may be necessary to give a saline injection into the rectum or subcutaneously. Iron and arsenic will probably be given, and the patient must have large quantities of fluid diet, such as milk, soups, etc. Between the attacks the general health must be attended to, and the bowels kept free. In bad cases stimulants may be necessary, but one must be very cautious in ordering alcohol, as a drinking habit may very easily be formed. The alcohol must be stopped as soon as the patient can do without it. The appropriate treatment of the various diseases which cause this condition will be dealt with as we discuss these diseases. Remember that it is not a disease of itself but merely a symptom or sign of disease.

*Metrorrhagia.*—This term is applied to an inter-menstrual discharge of blood from the uterus. It is practically the same as menorrhagia, only it does not occur at the usual time of menstruation. For instance a patient has a prolonged and profuse period, and then within a week or so of its ceasing the discharge commences again. This would be called metrorrhagia, or if you choose you could call it irregular and profuse menstruation, *i.e.* irregular menorrhagia. The causes are the same as of menorrhagia. In young women the most frequent cause is retention of a portion of the products of conception, In women above 35 the most frequent cause is malignant disease of the uterus. In every case in which it occurs in a woman over 35 one should look upon the cause as probably malignant disease and not be satisfied that it is not malignant until a very thorough investigation has been made. To decide the matter it would probably be necessary to have a microscopic examination of scapings from the endometrium. I wish to impress the importance of this upon nurses to make them realise how important it is for a woman who suffers from this to seek advice as soon as possible; therefore, if a woman over 35 complains to you of irregular and profuse menstruation, impress upon her the importance of seeking advice without delay. If she is suffering from malignant disease a delay of a few weeks may be fatal, as a cure is impossible unless an operation is performed early before the disease has had time to spread. Unfortunately, the majority of cases of malignant disease are not seen until it is too late.

*Dysmenorrhœa*—By this term is meant painful menstruation. Menstruation, as a rule, is accompanied with some pain and discomfort, but when this is exaggerated it becomes dysmenorrhœa. It is merely a matter of degree. As a rule it is due to one or more of the following factors, viz., a lowered state of the constitution causing a tendency to neuralgia; or an abnormal condition of the uterus, Fallopian tubes, or ovaries. Various forms, such as neuralgia, congestive, obstructive, ovarian and membranous have been separately described, but we shall not attempt to draw such fine distinctions. It is common in gouty and rheumatic constitutions, and in weak anæmic women, or those suffering from malarial poisoning. Such women have a strong predisposition to neuralgic attacks, and as any part which becomes congested is sure to be attacked with pain, you can easily understand why the pelvic organs are liable to be affected during menstruation. An examination of such a case will not reveal anything abnormal in the pelvic organs. The term menorrhalgia has been suggested as being more descriptive of this form than dysmenorrhœa.

By far the greater number of cases are due to abnormalities or disease of the uterus, Fallopian tubes, or ovaries. It is apt to occur when the uterus is imperfectly developed, *i.e.*, in what is known as an infantile uterus, and also in a uterus which has become abnormally small from superinvolution after labour; metritis, *i.e.*, inflammation of the muscular wall of the uterus, and endometritis, or inflammation of the mucous lining both cause it. The endometrium, when in a normal condition is not at all sensitive, but when it is inflamed it becomes markedly so. Displacements of the uterus, especially flexions or acute bendings, are apt to cause it. An ante-flexion, *i.e.*, a forward bending, is more liable to cause it than a retro-flexion or backward bending. The cause of the pain in these cases has been ascribed to the damming back of the discharge, but it is doubtful if this is the case. Uterine tumours (fibroids) cause it, especially those situated near the endometrium.

Disease or abnormalities of the Fallopian tubes is another cause. There is no doubt that the tubes actively participate in menstruation, and when there is any abnormality about them pain is apt to arise.

Ovarian dysmenorrhœa has been denied by some, but there can be no doubt that painful menstruation is often associated with enlarged and tender ovaries.

In some cases of dysmenorrhœa shreds of membrane, or it may be a complete cast of the uterus is expelled. This is known as membranous dysmenorrhœa. In some cases there is very little pain, but as a rule the suffering is great while the membrane is being expelled. We do not know the exact cause, but the condition probably results from a low form of endometritis. It usually occurs between the age of 20 and 30 years. The large majority of cases occur in married women, many of whom are sterile. Pregnancy does not seem to cure it.

*Intermenstrual Pain.—Middle pain*, or what the Germans call Mittelschmertz, may be conveniently considered along with dysmenorrhœa. It usually occurs about mid-term, and is not associated with a bloody discharge from the uterus. The patient suffers from very severe pain for a few days and then gets relief. It usually recurs very regularly.

The cause of it is often difficult to find, but there is no doubt that in some cases it is associated with pelvic tumour.

### Night-nursing in Mashonaland.

A REMINISCENCE BY A CORRESPONDENT IN RHODESIA.

FROM a lay point of view there is always a slight sense of eeriness and uncanniness in night-nursing. But if even in home hospitals we are assailed by reflections of this kind, they are intensified a thousandfold in those lonely up-country hospitals in the African veldt, where the town itself in which the hospital is built hardly counts more than half a score of years and the elements surrounding it are primitive and savage to a degree.

### THE OLD UMTALI HOSPITAL.

Few of the up-country hospitals in Rhodesia have their resident medical officer living in the building; his house is generally built some sixty or seventy yards away at nearest, and in the old Umtali Hospital in Mashonaland the one and only doctor lived in the town, about a mile and a half away from the hospital. No dispenser or secretary being kept, at night there was not a man near the place at all, except the patients, very few of whom were able to help the night nurse, a sharp attack of malaria rendering them very weak whilst at the height of the fever, and the moment they were at all recovered they went out of hospital. In the year 1897, just after the Matabele rebellion, there seemed no reason why the hospital folk should not be overcome by a surprise party of natives, and all the people in it murdered, long before help could have come to them. Umtali no longer exists, but for some eight or nine years it flourished gaily, and during this time nursing was carried on under many difficulties. The night nurse had no long united ward to be responsible for, where at a glance she could scan each bed, and hear the various breathings of the occupants, but the hospital was cut up into many small wards, opening out of each other, the scullery at one end, the night nurse's room at the other, and a "stoep" or verandah all round. It is one of the small peculiarities of South African doors that they rarely shut, and though provided with lock and key never lock. Moreover, the windows in those days were innocent of fastenings, and nothing would have been easier than for a native or Cape boy to slip in quietly and accomplish his purpose without the night nurse knowing anything at all about it unless she kept a very sharp look-out.

### THE NIGHT NURSE'S DUTIES.

Then again it is usual for the up-country hospitals to be built entirely for the white men; hence the wards for the natives were built far away at the end of the "compound" or enclosure, and if any of them were very ill it was necessary for the night nurse to visit them as quickly as possible, leaving the other portion of the hospital to its own devices. The lighting arrangements were of the most primitive kind; lamps were a rarity and candles had to answer for most ordinary purposes, great efforts being called for in the event of an emergency operation at night. The night nurse made her rounds with the aid of a lantern, it being the rule to go right through the hospital once in every half hour. One of her duties consisted in keeping an eye on the huts in which the day staff slept, as drunken men were apt to mistake the hospital for the B.S.A. camp, which was about half a mile off. The latter had to be tenderly conducted beyond the wire which enclosed the compound to ensure their safe departure. Another somewhat quaint and yet very real duty consisted in keeping the compound free of cattle, oxen, and horses straying around and disturbing both patients and day staff. The amount of work to be done by the night nurse was very variable, the treatment for malaria fever varying very much according to the doctor attending the case. In some instances the patient arriving with the "shakes" on him was put to bed at once, given a hot drink, and some seven or eight blankets put on the top of him. He was also given two hot-water bottles and a sweating powder, which if all went well produced a profuse perspiration at the end of about half an hour, necessitating a brisk rub down with hot towels, all clothes to be changed. This was an exceedingly lively half hour for both the patient and the nurse. Night nursing in old Umtali was a far more responsible thing than nursing in Rhodesia is now; the doctor being so far away a very close watch had to be kept over serious cases, so that at the slightest change for the worse he might be sent for in time. With regard to less serious cases more licence was allowed the nurse as to food, stimulants, and management generally than is possible where the medical officer lives on the premises. Also, when severe, the tropical diseases are much quicker in running their course, and changes in the condition of the patient come about very suddenly. There was never more than one night nurse, although in case of an emergency the matron had to be called. A thoughtful nurse, however, knowing the long, hot, arduous day that lay behind and in front of the matron, refrained from disturbing her well-earned rest.

### CALLING THE NATIVES.

Perhaps the most unpleasant duty of the night nurse was to call the natives at 5.30 A.M. In the rainy season yards of mud and water had to be paddled through in order to get to the "boys' kyre" in which some 10 or 20 natives slept, rolled up in their blankets on the floor, in undistinguishable bundles, turning out with many yawns at the request of the "nightie missisie"—a request, when gentle measures failed, urged at the end of a broom-handle by the much-tried Rhodesian night nurse, who finally retired to her rest in a little thatched hut, half-way up the hill, placed there to ensure quiet and some degree of coolness.

### District Nursing.

CONFERENCE OF SUPERINTENDENTS (BY OUR OWN CORRESPONDENT).

In February last year the first conference of the superintendents of the North of England was held at the Central Home, Liverpool, and it was then decided that, as far as possible, such a conference should take place annually. This year, through the kind arrangement of Miss Blower and Miss Heygate with their committees in Manchester, the second conference was held on Thursday, February 20th, at Salford. The superintendents from Manchester, Liverpool, Bolton, Blackburn, Leeds, Rochdale, Newcastle, Sunderland, and Birmingham journeyed to Manchester to take part in the discussion on the various subjects chosen by the sub-committee.

At 12 o'clock Canon Hicks welcomed the visitors in the name of the committee, and in well-chosen and effective words spoke of the importance of the work of district nursing, and the great service done to all the poor and sick and suffering by Mr. William Rathbone's generosity and wisdom in founding and organising the work.

Luncheon was served at one o'clock, and afterwards the conference proper began in the nurses' cosy sitting-room, which was so arranged as to make everyone feel very much at home.

Letters of apology for non-attendance were received from 10 superintendents, thus making the number rather fewer than last year.

The minutes of the last meeting were read and confirmed, and the committee were re-elected, with power to add to their number. At present the committee consists of Miss Blower, Miss Heygate, Miss Wilson, Miss Barlow, Miss Andrew, and Miss Rogers.

Miss Rosalind Paget, who presided, in opening the conference, expressed her pleasure in again being present, and spoke of the benefit which she herself had received from the discussions last year.

The first subject was " Giving of Relief : how it should be organised, the co-operation of the philanthropic amateur, and the advantages of the Charity Organisation Society."

Miss Wilson, Liverpool, introduced it in a concise and suggestive paper. The views of superintendents from various centres were heard, and many valuable suggestions were given, but all were of opinion that the giving of relief should be quite apart from the work of the nurse; that it is most necessary that there should not only be milk allowed in cases of poverty and weakness, but that there should also be depôts where well-cooked food can be obtained in connection with, but apart from, the nurse's visits; and that through some such organisation many valuable lives might be saved and the days of suffering greatly lessened.

The following resolution was proposed by Miss Heygate, Salford, seconded by Miss Barlow, Leeds, and unanimously carried:—

" That the superintendents present consider that the present system of giving relief is not perfect; that there is a great danger of the nurse being considered the almoner, which is not desirable, and that it would be of the greatest assistance to the work if a committee could be formed in connection with every association, which would deal with the giving of relief apart from the work of the nurse."

Many superintendents spoke of the advantages to be obtained from the co-operation of the philanthropic amateur and the encouragement it was to a superintendent when members of committees and others interest themselves in the patients unprofessionally and independent of the nurse's visits.

Miss Walker, Bolton, read an interesting paper on the difficulties of obtaining " Extra Nursing Help," the best sort to be obtained, and the difficulties of having untrained women as helps in certain cases. The experience of most of those present went to show that partially-trained help was unsatisfactory, and it was unanimously agreed that where there are a large number of nurses in a home an extra nurse is a most desirable thing. Many admitted the advantages of having some untrained but dependable women who can work under the nurse for the chronic patients, thus giving the nurse more time for her acute cases and enabling her to undertake more.

Miss Barlow, Leeds, spoke of the use and abuse of nursing appliances and the difficulty there was in obtaining the supply to meet the demand in busy districts. She advocated a small charge being made whenever possible in order that the friends of patients might realise the value of such comforts and form a fund for buying new ones.

The training of nurses in district work and the amount of training which might be given was ably dealt with by Miss Mills, Liverpool, and Miss Chadwick, Blackburn.

Miss Mills spoke of the possibility of continuing the training of the nurses through the constant supervision and advice of the superintendent in visiting with the nurse, and Miss Chadwick suggested that nurses should receive a certain amount of training from the superintendent in housekeeping and book-keeping and be brought into contact with the members of committee, so as to give them experience which would help them in the future either as superintendents or in solitary districts.

Several interesting subjects were suggested for next conference, such as, the advantages of " daily visiting " nursing and the advisability of carrying on private and district nursing work in the same homes.

It was also suggested that the doings of district nurses should be published regularly in a periodical, so as to form a connecting link for Queen's nurses scattered over the country with few opportunities of meeting.

Miss Heygate proposed, and Miss Wilson seconded, a vote of thanks to Miss Paget, which was warmly and enthusiastically given. Miss Paget acceded to the unanimous request that she should take the chair at the conference next year.

After tea the various members separated with many expressions of pleasure at having had the opportunity of seeing the delightfully arranged Home at Salford, and of meeting friends and acquaintances of past years. Miss Heygate and Miss Blower must have been gratified at the many expressions of gratitude for their kind thought for all their guests.

### To Nurses.

**We invite contributions from any of our readers, and shall be glad to pay for " Notes on News from the Nursing World," or for articles describing nursing experiences, or dealing with any nursing question from an original point of view. The minimum payment for contributions is 5s., but we welcome interesting contributions of a column, or a page, in length. It may be added that notices of appointments, entertainments, presentations, and deaths are not paid for, but that we are always glad to receive them. All rejected manuscripts are returned in due course, and all payments for manuscripts used are made as early as possible after the beginning of each quarter.**

### Everybody's Opinion.

[Correspondence on all subjects is invited, but we cannot in any way be responsible for the opinions expressed by our correspondents. No communication can be entertained if the name and address of the correspondent are not given as a guarantee of good faith, but not necessarily for publication. All correspondents should write on one side of the paper only.]

### BRIGHTON UNION INFIRMARY.

"ELIZABETH SMITH," who has just been appointed nurse at Brighton Union Infirmary, writes: I wish to say that I was not on the staff of the North London Hospital, but only stayed there for my own convenience.

### A DISTRICT NURSE'S APPEAL.

"NURSE ANNIE" writes from Mexboro', Rotherham: Very many thanks for publishing my "Want." I have had such an enormous number of answers that it is impossible to answer them except through your columns. I gratefully thank all those who have offered THE HOSPITAL to me, and beg to say that I accepted the first offer sent.

### NURSES AND BEDSORES.

"B. L." writes: The controversy of nurses on the paper headed "The Nurses' Don't," has much amused me, and I am hoping that some time you may have a series of "The Nurses' Do." I have also taken much interest in the essays from nurses upon bedsores. Although I have been an invalid for some years now, before I became so, and when trained nurses were comparatively few, being very fond of the work, I was constantly nursing sick friends, and I never had a bedsore of any kind. This I attribute to using a large basin of cold water renewed daily, containing a few drops of carbolic, or Sanitas, or home-made Condy. My personal experience now is equally happy, and I have been confined to my bed for eight months. I have been a reader of your paper for many years, and after perusing it I send it first to two friends, and then to two nurses.

### LOW SALARIES.

"THE BROOM OF THE WAVE" writes: I should like to draw your attention to the ridiculously low salaries offered to fully-trained nurses in the HOSPITAL. Surely a "Sister," for instance, who has a three years' certificate and is required to take charge of a ward and operating theatre is worth more than £25 a year—a sum that a good housemaid can obtain easily. What inducement then is there for a woman who, apart from her love of the work, has her living to earn to become a nurse, if at the end of three years' training, and having possibly paid a premium and received very little pay during training, she can only earn £25 per year? At some hospitals the premiums are very high, and this may mean years of saving to some who wish to enter for training. I know of one hospital where the premium is £50. I think nurses are greatly to blame for working for such small salaries. Of course a great many can afford to do so; others who have friends dependent on them cannot. For these it is very hard. With so much competition going on they have often to accept these small salaries or starve; £25 a year is barely sufficient to provide a nurse with ordinary comforts and a yearly holiday. How can a nurse save up for the old age which as a rule comes more quickly to nurses than any other class of women-workers? It is a well-known fact that nursing takes years off a woman's life. I should like to hear the opinions of some of your correspondents on the subject.

### "DISGRACEFUL NEGLECT AT MILE END."

"J. HARLEY BROOKS, M.D., Medical Superintendent at Mile End Infirmary, Bancroft Road, N.E.," writes: The paragraph in your last issue headed "Disgraceful Neglect at Mile End" has been brought under my notice, and as it is of a rather damaging character, I must ask you to allow me the opportunity of controverting it in your pages. I do not know to whom you made the inquiry which was ignored—not to me at all events, and your charge of neglect comes as a surprise to me. In the first instance, the statement that "of the seven nurses who caught small-pox at Mile End Infirmary not one had been vaccinated" is not true, for each had been vaccinated in childhood, as had all the rest of the nurses. The Mile End Infirmary is a Poor Law institution for the treatment of the sick poor of the parish and not a small-pox hospital, and the nurses are engaged for the nursing of ordinary cases of sickness. They come first as probationers and undergo a course of training extending over three years. At their appointment no religious tests are applied, nor has it hitherto been the custom to require them to be vaccinated or revaccinated. The law at present recognises the conscientious objector, and revaccination is not compulsory in this country. However, when small-pox became prevalent in London, I made an offer of revaccination to the infirmary staff, and a large majority availed themselves of my offer, but a few declined, a course which was perhaps foolish on their part, but nevertheless within their legitimate rights. Seven of these have unfortunately paid for their folly by contracting small-pox. The disease broke out in the infirmary on January 25th, and the infection has since been traced to a case which was admitted on the 15th and died next day, the true nature of which, however, was not recognised till the outbreak in the wards on the 25th. Meanwhile the contagion had been latently at work and the seven nurses were almost simultaneously attacked with small-pox. Of these seven nurses only one came into actual contact with a case of small-pox recognisable as such by the rash appearing. This nurse was revaccinated next day, went off duty the following day, and was removed the succeeding day suffering from small-pox. The seven nurses contracted the disease from the unsuspected case, none of them by direct contact with it, but from associating with the nurses who dealt with the case. The mischief was all done in the latent or incubation period and before the actual outbreak of small-pox in the wards. I quite agree with you that revaccination is a proper precaution to be taken by all nurses, and I not only made the offer, but also very strongly urged it. That being so, I fail to see any disgraceful neglect on my part. I am glad to say that all the nurses have got on well, and no loss of life is likely to be added as a further penalty for their folly.

### DISTRICT NURSING IN THE WEST OF ENGLAND.

"NURSE P." writes: Whilst district nursing in the West of England I was surprised at the simpleness of the cottage folk. At first the old superstitions which the people believed in amused me greatly. An old woman with a bad leg frequently told me it was all because she had been overlooked, and that therefore the cause of her ailment was the troublesome "evil eye." I looked quite serious after such a remark, lest the patient should feel hurt. Another patient, on being asked if her cramp were better, replied, "Oh, yes, much better since I have kept a cork under my pillow." She would ask me gravely if I believed in the treatment. Sometimes a small potato was carried in the pocket as a cure for rheumatism. One morning I found a bottle of cold water tightly corked in the bed as a cure for bed-sores, whereas the poor patient was lying in saturated sheets, which no one ever thought of taking away. I had a very funny old couple in my district. The patient was the old man, who had met with an accident whilst driving; this had caused him to be quite bed-ridden. Whilst I made him comfortable he used to storm frightfully, telling me I was not fit to be a doctor, and that he would tell everyone so. His spouse resented such behaviour on his part, and told him he was not to talk like that to the lady, or else she would give him a good smack, and she would carry out her threat, saying in an apologetic manner, "He never was right, miss, it's what the Lord blessed him wi'." It seemed useless for me to say that she must not smack him, for she often greeted me in the morning with "I have been smacking him right well, miss, I couldn't help it." At last I frightened her by saying that if he died and any marks were found on him, she would probably be accused of manslaughter. On another occasion I found a patient with treacle plasters on as a remedy for gathered breasts. At first all this presented a humorous aspect to me; then I thought it was sad that in this enlightened age there should be so much ignorance. I have done district nursing in other parts of the country, but have never found any cottage folk nearly so simple as those in the county of Somerset. Of course, one frequently gets told that the patient has conjunction of both lungs, an ulster in the throat, pewmonia in the head, and brownkitis which affected every limb. These mistakes, however, are pardonable when compared with the silly superstitions of Somerset and Devon.

### Appointments.

[No charge is made for announcements under this head, and we are always glad to receive, and publish, appointments. But it is essential that in all cases the school of training should be given.]

Birmingham and Midland Skin and Urinary Hospital.—Miss Mary T. Castle has been appointed matron. She was trained for three years at the Bristol General Hospital.

Cranleigh Village Hospital.—Miss Ellen Longstaffe has been appointed matron nurse. She was trained at the London Hospital, E., and has since been matron for twelve months at the Victoria Hospital, Worksop.

Hospital for Women, Liverpool.—Miss Scott Cavell has been appointed assistant matron. She was trained at the Metropolitan Hospital for three years. She has since been charge nurse at the Fountain Fever Hospital, Tooting. Ward sister at Poplar and Stepney sick asylum, temporary night superintendent at Bethnal Green Infirmary, and since May, 1900, has been night superintendent at Hendon Sick Asylum.

Prudhoe Memorial Convalescent Home, Whitley, Northumberland. — Miss Helena A. Gomme has been appointed lady superintendent. She was trained at the Hospital for Sick Children, Great Ormond Street, and at the Glasgow Royal Infirmary, afterwards acting as night sister and sister at the Children's Hospital, Glasgow. For seven years she worked in connection with the Nurses' Co-operation, 8 New Cavendish Street, London, as a supernumerary nurse.

Royal Infirmary, Halifax.—Miss M. R. Manley has been appointed sister. She was trained at Huddersfield Infirmary and at the Fever Hospital, Middlesbrough, and has since been sister of the children's ward at Macclesfield Infirmary.

St. Olave's Union Infirmary.—Miss Alice Driscoll has been appointed charge nurse. She was trained at St. Olave's Union Infirmary, where she has since been nurse. She has also been head nurse at Uttoxeter Union Infirmary, and ambulance charge nurse at Gore Farm Hospital.

Somerset Hospital, Capetown.—Miss Georgina Hopper has been appointed matron. She was trained at Addenbrooke's Hospital, Cambridge, and St. Mary's Hospital, London. She has since held the following appointments Charge nurse of the women's ward at the Royal Mineral Hospital, Bath; superintendent of nursing home at Worthing; superintendent of the district nurses during the typhoid fever epidemic at Worthing in 1893; in charge of the women's medical and ophthalmic wards at the East Suffolk Hospital, Ipswich; in charge of men's accident and surgical wards in the same institution; and matron at Bushey Heath Hospital.

Stafford Union Infirmary.—Miss Rose Anne Grant has been appointed superintendent nurse. She was trained for three years at St. George's-in-the-East Infirmary, London, where she was afterwards staff nurse for one year and sister for two years.

Steyning Union Infirmary.—Miss Ada B. Clarke has been appointed superintendent nurse. She was trained for three years at the Birmingham Infirmary, and at the City Hospital, Birmingham. She has been charge nurse at Yarmouth Fever Hospital; home sister and night superintendent, and subsequently assistant matron at the Union Hospital, Stockport. Miss Clarke holds the L.O.S. certificate.

Wath, Swinton, Greasborough, and Rotherham Joint Fever Hospital.—Miss May L. Macdonald has been appointed matron. She was trained at Yeatman Hospital, Sherborne, and has since been charge nurse at Ham Green Hospital, Bristol.

Westmeath Infirmary, Mullingar, Ireland.—Miss Julianne Kelly has been appointed nurse. She was trained at Jarvis Street Hospital, Dublin, and has since been doing private nursing in England.

Winchcombe Cottage Hospital, Gloucester.—Miss Daisy L. Vernon has been appointed nurse-matron. She was trained at Sheffield General Infirmary, and has since been head nurse at Bishop Auckland Union Hospital, Durham; sister of the women's ward at Essex and Colchester Hospital; sister of the men's medical, surgical, and ophthalmic wards, East Suffolk and Ipswich Hospital; and matron at the Hospital for Skin and Urinary Diseases, John Bright Street, Birmingham.

### Death in Our Ranks.

The Army Nursing Service. — The death of Miss Dorlinda Bessie Hyland at Bloemfontein, from enteric fever, took place on February 14th. Sister Hyland, who was a native of Lancaster, and a member of the Army Nursing Service, had been nursing in South Africa for nearly two years, latterly in the Concentration Camps. Prior to this she was engaged for eighteen months in the plague district of India.

The Matron of Gateshead Fever Hospital.—We also regret to learn of the death of Miss Margaret Pender, matron of the Gateshead Corporation Fever Hospital, from typhoid fever. Miss Pender, who was 41 years of age, had been matron at Gateshead for eight years, and possessed the entire confidence of the Gateshead Town Council. Her illness was of several weeks' duration, and at one time it was hoped that she would recover.

The Matron of Walsall Hospital.—We regret to hear of the death, from pneumonia, of Miss Georgina Harriot Sked, matron of Walsall and District Hospital. Miss Sked who was 37 years of age, was the younger daughter of the late Mr. James Sked, of Rockferry, Cheshire, and was trained at the Royal Infirmary, Edinburgh, and the Liverpool Royal Infirmary. She subsequently, after being three months at Myrtle Street Children's Hospital, became assistant matron at the Royal Albert Edward, Wigan, then matron of the Isle of Wight Infirmary and County Hospital, Ryde, Isle of Wight; and on July 9th, 1901, took up the duties of matron of Walsall and District Hospital. In this position she quickly earned great esteem, and her death is much lamented by all who were associated with her. The funeral took place at Bebington Cemetery on Friday.

### Presentations.

Rockferry Nursing Institute. — Miss Helen Hulse, who is leaving England for Sydney, where she goes to be married to Captain G. B. Whiting, has been presented by the matron of the Rockferry Nursing Institute, to which she was attached, with a brass drawing-room clock; by her fellow nurses with a full set of toilet brushes with silver initials; and by the maids of the institute with a photographic screen.

### The Importance of Position in Accident and Disease.

Lecture by Dr. Robert Bowles.

On Thursday last week Dr. Robert Bowles lectured to the members of the Royal British Nurses' Association at 10 Orchard Street, on "The Importance of Position in Accident, Disease, etc." The lecture, Dr. Bowles explained, would be more truly described as a series of demonstrations, and he was aided in his remarks by diagrams, a sheep's head and lungs, and a boy to illustrate the various positions. It was, the lecturer said, in 1856 that he became enthusiastic about the treatment of cases of drowning; he was then living at Folkestone, and was sent for frequently to attend shipwrecked sailors. The average London doctor, he continued, hardly saw a case of drowning once in 20 years, since they were dealt with by the Royal Humane Society; but from his large experience of such cases he was able to lay it down as a general rule that the patient should never be allowed to lie on his back, but always on his side or face downwards. The same rule applied, generally speaking, to cases with which they as nurses had frequently to deal, viz., epilepsy, apoplexy, hæmoptysis, suffocation in cases of phthisis, and choking in old people. In cases of drowning the water and mucus which was stopping up the air passages must be squeezed out by firm pressure on the back of the patient.

With the aid of the sheep's head Dr. Bowles demonstrated the position of the tongue when the head is turned backwards, as in lying on the back, showing that the tongue blocked up the entrance to the windpipe. On several occasions he had stopped a fit of choking in a patient by pulling up the tongue or turning him over on his side. If there was a rattling in the throat, the patient must never be allowed to lie on his back, but on his side; and, in cases of lung disease, on the side affected, so that the strong lung should do the work of both.

At the close of the lecture several questions were asked by the nurses: one wanted to know which side the patient should lie on when there was no disease; another, whether there was not some risk attached to treating a case of apoplexy in the way recommended, when it was not known which side was affected. A third asked how an unconscious patient should be fed; a fourth, whether, if fluid food got into the lung, it could be pressed out; and a fifth asked whether the same methods would apply to trying to revive an apparently still-born child.

Dr. Bowles replied (1) the right side was the one to be chosen unless there was any reason to the contrary; he had observed that healthy people usually turned on that side in sleep, and he thought it was because the weight of the liver was not then felt. But if there was disease of the lungs, the patient should be placed on the bad side. (2) When the strumous breathing was done away with by his method, it was then possible to find out which was the paralysed side by observing which arm fell as a dead weight; then that side should be put downwards. There was no risk, but the question, Dr. Bowles observed, was a very important one. (3) Turn the unconscious patient on his side. If fed on his back, the fluid would go into the lungs. Holding the nose would not obviate this. (4) The fluid could be squeezed out if the method was applied immediately. (5) The same method could not apply to the case of an apparently still-born child, because the lung did not exist.

A very hearty vote of thanks for what all the nurses agreed had been a most interesting lecture was accorded, on the motion of Mr. Alfred Cooper, who was in the chair.

### For Reading to the Sick.

"THY WAY, NOT MINE."

Take Thine own way with me, dear Lord,
Thou canst not otherwise than bless;
I launch me forth upon a sea
Of boundless love and tenderness.

I will not fear Thee, O my God!
The days to come can only bring
Their perfect sequences of love,
Thy larger, deeper comforting.

Within the shadow of this love,
Loss doth transmute itself to gain;
Faith veils earth's sorrows in its light,
And straightway lives above her pain.

*Jean Sophia Pigott.*

God does not treat His sufferers like children who are simply to be petted with soft words, and patted with soft hands till they forget their griefs. He deals with them as men who are capable of knowing the meaning, the explanations, and the purposes of the troubles that come to them. And so He gives them His great truths of consolation. What are those truths? Education, spirituality, and immortality, these seem to be the sum of them. Is there no consolation in these great thoughts? They do not take your sorrow off; and oh, whatever be your suffering, I beg you to learn first of all that not that, not to take your sorrow off, is what God means, but to put strength into you, that you may carry it as the tired man, who has drunk the strength-giving river, lifts up his burden by the river-bank and goes singing on his way. Be sure your sorrow is not giving you its best unless it makes you a more thoughtful man than you have ever been before, unless it opens to you ideas that have before been unfamiliar; mostly these three ideas—education, spirituality, immortality. Those ideas are the keys of all the mysteries of life, and so the gateways to consolation. And it is wonderful to see how, just as soon as a man is really crushed and sorrowful, God seems by every avenue to be offering those great ideas for that man's acceptance. He seems to write them on the sky, to whisper them from every movement of the commonest machinery of life, to fill books with them that never seemed to know anything of them before, to make the vacant house and the full grave declare them. You are a child of God whom He is training. You have a soul which is your true value. You are to live for ever. Know these truths. By them triumph over the sorrow that He cannot take away, and be consoled.

*Phillips Brooks.*

I laid it down in silence,
This work of mine,
And took what had been sent me—
A resting time.
The Master's voice had called me
To rest apart;
"Apart with Jesus only,"
Echoed my heart.

. . . . . . .

We are but *under*—workmen,
They never choose
If this tool, or if that one
Their hands shall use;
In working or in waiting,
May we fulfil
Not ours at all, but only
The Master's will.

*S. M. E.*

### Off-duty Letter.

### A WOMAN'S PLAY AT THE LYRIC THEATRE.

I had been looking forward with much interest to seeing "Mice and Men" because of its authorship. The reproach has so often been levelled at women, that although they can succeed in most things scarcely a single woman has managed to write a really clever, well-constructed play, that I was curious to appraise for myself the merits of a drama which has so thoroughly hit the public taste as Mrs. Madeleine Lucette Ryley's production. The house was crammed; the booking is, I am told, extremely heavy; and as I emerged from the theatre after the performance I heard on all sides exclamations: "What a pretty piece!" "Wasn't it sweet?" "Isn't he a dear?" and commendation of an equally ladylike and correct description. Moreover, these amateur critics are right, "Mice and Men" is undoubtedly a pretty piece, it is very "sweet," and the self-sacrificing hero is a "dear," but the fact that such a play should in a sense have taken the town by storm does not say much for the elevation of dramatic taste to which the general public is supposed to have been educated.

As to the writing, here and there are clever remarks and telling phrases, more especially in the first act, which is by far the best and full of possibilities; but there is a lack of sustained interest, and men and women alike suffer from a lack of spontaneity and a certain stiffness which are, perhaps, accentuated by the old-fashioned English of the period, 1786. For the most part the characters are puppets, who do the correct thing when the creator pulls the string; but Mrs. Ryley has yet to discover the wire which will vivify them into life. However, in one direction, at least, we owe the authoress a debt of gratitude. The clean wholesome tendency of the play and the manly way in which Captain Lovell determines to shake off former shackles — deserve nothing but praise. The mere fact that the theatre-going public flock to the Lyric, knowing that they will witness only a simple story of everyday life, makes one hopeful that "problem" plays, as they are called, have, from their very nastiness, commenced to lose the power to attract.

Respecting the scheme which "gangs a-gley," Mark Embury is a scholar, a scientist, and a philosophist who has arrived at middle age without any desire for matrimony. At this period he conceives the brilliant idea of training up a wife in his own theories, which include running about barefoot in the garden of his Hampstead residence. Having added a housekeeper to his modest staff, he sends for eight or nine girls from the Foundling Hospital, and selects one whose outward appearance pleases him. She is named "Little Britain, after the locality where she was found, but he elects to call her "Peggy." Having said farewell to her companions, the *protégée* is bidden to occupy her mind with recounting her multiplication table till her guardian returns. The recitation has progressed but a little when a young and handsome captain, Mark Embury's nephew, arrives—through the window. He has returned to look for a miniature of the lady of whom he is enamoured (the wife of Mr. Embury's greatest friend, Mr. Goodlake), which he had dropped during a stormy interview with his uncle earlier in the day. Captain Lovell teaches Peggy something different to multiplication table, and the first nail is driven into the coffin of the celebrated scheme. Being sent for anon by his uncle, he promises to reform his ways, receives his uncle's forgiveness and the lost miniature, and goes off to Ireland. As the curtain falls the guardian is seen giving his ward her first writing lesson.

Two years are supposed to have elapsed before the second scene in the "living" apartment at Mr. Embury's. The plan appears to be working well, the scholar has grown into the lover and has thriven under the change, and, hopeful that Peggy loves him, he makes an attempt to acquaint her with the reason why he adopted her, of which she has hitherto been kept in ignorance. But interruptions come, and meanwhile the Captain is expected from Ireland. Mrs. Goodlake, who is determined not to lose a handsome admirer, persuades Peggy that Captain Lovell loves a dear friend of hers, and under pretence of sending a message from the lady asks Peggy to deliver a few lines secretly to the Captain making an assignation with him at a coming masquerade ball. As a recompense, Mrs. Goodlake promises to take Peggy also to the ball, to lend her a fancy dress, and to keep the escapade a secret. Peggy carries out her part of the bargain, but the Captain, determined to have no more to do with the lady, throws the *billet doux* into a dress basket, which is being sent to Mrs. Goodlake, hoping it will thus be given back to the writer, and makes himself agreeable to Peggy.

The third act takes place in the ante-room of Belsize House during the ball. Mrs. Goodlake in vain pleads with the Captain to restore her to the former place she held in his affections; he refuses to do so, and shows that his allegiance has been transferred to Peggy. But the young lady, believing that he only follows his uncle's wishes in making love to her, affords him no encouragement, and the troubled waters are still further stirred by the appearance of Mr. Goodlake and Mark Embury. The former has found his wife's letter, which the Captain threw into the basket, and has followed her to the ball. Peggy, believing Mrs. Goodlake's statement that if she tells the truth the uncle will disown his nephew, takes the burden of all the blame upon herself, says that Mrs. Goodlake wrote the love-letter for her; that Mrs. Goodlake came solely to escort her, and, having seen the husband triumphantly lead away his wife and Captain Lovell depart, she beseeches her guardian to kill her, if he wishes, "she is so wicked," but to forgive her. This necessarily leads to an avowal on his part, and she promises to marry him as soon as he likes.

The last act shows the exterior of South Cottage, the home which has been prepared for Peggy's married life. She has never seen the interior, although she is to be wedded next day. Everything is complete, even to mahogany chairs, a harpsichord, and "six dozen of everything," and the housekeeper hands over the keys, but says that the future mistress is to wait to be shown round by her future husband. After some rather dolorous lovemaking between guardian and ward, the latter promises, at the request of Mr. Embury, to ask the Captain to stay for the wedding, which he had not intended to do. When the gallant officer comes in answer to a summons, the request is made, and the letter is read, wherein Mr. Embury states that, knowing Peggy loves George Lovell, and having ascertained by observation that her love was returned, the writer resigns his claim and wishes them every happiness together. He adds that South Cottage is to be Peggy's wedding portion. Then as the happy couple enter the front door, Mark Embury crosses the stage, looks sadly at the house, whence comes sounds of music, and passes through the gate into the outside world.

The part of Mark Embury is exactly suited to Mr. Forbes Robertson. Even the old-fashioned dress becomes him. He looks the scholar to the life, his voice gives due emphasis to his words, and in the last act his resignation is apparent in his very features. Miss Gertrude Elliott is very fascinating in her pretty Foundling Hospital dress, and her naïveté in the earlier scenes is not overdone; but the situation in the later portions of the play is more trying, and both Mr. Ben Webster and Miss Elliott apparently find it difficult to be thoroughly at ease. Miss Mary Rorke makes a comely housekeeper; Mr. Luigi Lablache a handsome and hearty Roger Goodlake; and Mr. William Farren, jun.'s, impersonation of Peter, Embury's servant, deserves special mention. The last is a most finished and characteristic sketch of a rather cantankerous, rather dirty, faithful old man who cares little for anyone or anything but his master and what he considers his duty. The scenery, especially of the heights of Hampstead, is thoroughly good and appropriate.

### Notes and Queries.

**The Editor is always willing to answer in this column, without any fee, all reasonable questions, as soon as possible.**
**But the following rules must be carefully observed :—**
**1. Every communication must be accompanied by the name and address of the writer.**
**2. The question must always bear upon nursing, directly or indirectly.**
**If an answer is required by letter a fee of half-a-crown must be enclosed with the note containing the inquiry, and we cannot undertake to forward letters addressed to correspondents making inquiries. It is therefore requested that our readers will not enclose either a stamp or a stamped envelope.**

*Crepe Bandages.*

(192) I should feel obliged if you will give me the address of the makers of crepe bandages.—*Nurse Frances.*

The three leading makers are Messrs A. de St. Dalmas & Co., Leicester; Mr. M. Sello, 12A Finsbury Square, London, E.C.; and Mr. Vincent Wood, 4 Albion Place, Blackfriars, S.E.

*Nurse's Cloak.*

(193) Kindly tell me where I can get a pattern of a nurse's cloak with sleeves. My new one is spoiled.—*Nurse F.*

Write to the various pattern firms; or unpick an old cloak of your own, carefully iron the pieces smooth, and you will then not only have the pattern of a cloak, but of a cloak that fits you.

*Handbook.*

(194) Can you recommend a suitable handbook on home nursing for the matron of a boys' school who is not a trained nurse?—*Sphinx.*

"The Mother's Help and Guide to the Domestic Management of Her Children," by P. Murray Braidwood, M.D. Price 2s. Published by the Scientific Press.

*Baldness.*

(195) My wife is becoming bald, and the use of hair-restorers has failed to do any good. Is there any hospital in or near London for such cases, as I think it may be a disease of the scalp or of the nerves?—*F. W. E.*

The case is one for medical advice, and eligible for treatment at any skin hospital. The Hospital for Diseases of the Skin, 52 Stamford Street, Blackfriars, S.E., might be suitable.

*Home for Infant.*

(196) Is there any home where a lady can place her infant from birth?—*Mrs. F.*

There is no public institution, but if you advertise in THE HOSPITAL you will get plenty of suitable answers.

*Home for Paralytic.*

(197) Can you tell me of a hospital where a paralysed man could be received? His friends would pay 15s. a week.—*A. M. W.*

The National Hospital for the Paralysed, Queen Square, Bloomsbury, W.C., might be able to help you.

*Consulting Physician for Consumption.*

(198) Can you tell me the best consulting physician for consumption?—*Nurse May.*

We never recommend individual practitioners.

*South Africa.*

(199) I have travelled much and had 15 years' experience. Will you tell me if there would be any risk in going to South Africa on my own account, or would America be better?—*Sister.*

It would be most unwise to go to either of the countries you name, unless you have enough capital to live on for at least a year, pending regular employment.

*Hospital Training.*

(200) I wish to be trained as hospital nurse. I am a domestic servant. Will you kindly tell me if this will prevent my acceptance as probationer in an infirmary?—*E. J.*

Some institutions prefer domestic servants as probationers. See "The Nursing Profession: How and Where to Train."

A young friend of mine, aged 16, is anxious to become a nurse. Will you tell me if she is too young for a children's hospital, and where she could apply?—*E. M. J.*

She is too young for any hospital.

Are there any children's hospitals where probationers are trained without premium, and what is the age limit for entering?—*W. A. H.*

Yes, see "The Nursing Profession: How and Where to Train" for lists. The age limit varies. At some hospitals probationers are accepted as early as 20 and as late as 35 years of age.

Is the Victoria Hospital, Folkestone, a recognised training school for nurses, and are the three years' certificate awarded by that institution regarded as full nursing qualification?—*Paula.*

Whilst the Local Government Board has laid down general rules as to what constitutes "a recognised training school for nurses," it has not published a list of schools so recognised; it is, therefore, always advisable in cases of doubt to write direct to the Medical Inspector for Poor Law Purposes, Local Government Board, Whitehall, S.W., and ask if the training at the school in question is recognised as qualification for a superintendent nurse. The institution you refer to is a small hospital of 37 beds.

1. Will you kindly tell me if my being ruptured would prevent me becoming a hospital nurse? 2. Is the training in an infirmary as good as that given in a hospital?—*Would-be Nurse.*

1. This depends upon circumstances only to be decided by your medical examiner. 2. "Infirmary" and "hospital" are names applied indiscriminately to the same kind of institution. The value of the training depends upon the particular infirmary or hospital at which the nurse is trained.

Please tell me if there is any hospital in Aberdeen where a probationer of 21 would be trained without premium? I should prefer maternity training if possible.—*J. D.*

Apply the Royal Infirmary, Aberdeen. You had much better be trained in general nursing first, and then in maternity afterwards.

I am devoted to children, and am very anxious to be trained as a children's nurse, but as I am 35 years of age several hospitals have refused me. How can I possibly be trained?—*A. C.*

There are a large number of children's hospitals in the provinces as well as in London. Write to them all (see "The Nursing Profession: How and Where to Train" for lists), and explain your position fully.

I am a domestic servant, not very well educated. Will you tell me if it would be of any use applying to a children's hospital or to an infectious disease hospital for training as nurse?—*E. W.*

If you are strong and intelligent, some of the Poor Law infirmaries might be glad to train you. Write to the matron of such institutions as seem suitable. You will find out all about them in "The Nursing Profession: How and Where to Train."

I have a very great desire to become a nurse, but I am only seventeen. Will you kindly tell me what age I must be before I can be trained, and where is the best place to go for training?—*E. H.*

You should be twenty-three before you begin to train, but you can employ the intervening years very profitably by making yourself proficient in domestic duties and cooking.

*Homes.*

(201) Will you kindly tell me of any home for incurables which would be advisable for a female surgical case, a native of Wiltshire, and where no payment is necessary?—*C. P.*

You give no particulars. The case may be eligible for one of the charities provided for poor gentlefolk (for which see "Burdett's Hospitals and Charities") or for the workhouse infirmary.

Mrs. E. W. would be much obliged if the Editor could recommend any doctor's home for a young lady, an uncertified mental case.

We cannot recommend private persons.

I am a widow and a nurse, and I should be most grateful if you could tell me of a home in which I could place my son, twelve years of age. He has epileptic fits, which are now becoming more frequent.—*C. M.*

Write, giving full particulars, to the secretary, National Society for Employment of Epileptics, 12 Buckingham Street, Strand, W.C.

Will you kindly tell me how I can best make known that I receive convalescents after infectious diseases? I am a fully-trained nurse and my house is healthily situated near the Downs.—*E. P.*

A standing advertisement in a medical or nursing journal would be the best way of drawing attention to your home and of keeping it in the memory of doctors and nurses.

Can you tell me of a home or sanatorium in a bracing inland place for a girl recovering from chest trouble? She is able to pay a very small sum only.—*H. J.*

See the list of convalescent homes in "Burdett's Hospitals and Charities." You give no particulars, and the choice for such a case must be made with care.

**Standard Books of Reference.**

**"The Nursing Profession: How and Where to Train." 2s. net; post free 2s. 4d.**
**"Burdett's Official Nursing Directory." 3s. net; post free, 3s. 4d.**
**"Burdett's Hospitals and Charities." 5s.**
**"The Nurses' Dictionary of Medical Terms." 2s.**
**"Burdett's Series of Nursing Text-Books." 1s. each.**
**"A Handbook for Nurses." (Illustrated). 5s.**
**"Nursing: Its Theory and Practice." New Edition. 3s. 6d.**
**"Helps in Sickness and to Health." Fifteenth Thousand. 5s.**
**"The Physiological Feeding of Infants." 1s.**
**"The Physiological Nursery Chart." 1s.; post free, 1s. 3d.**
**"Hospital Expenditure: The Commissariat." 2s. 6d.**
**All these are published by the SCIENTIFIC PRESS, Ltd., and may be obtained through any bookseller or direct from the publishers, 28 and 29 Southampton Street, London, W.C.**

### Travel Notes.

By Our Travelling Correspondent.

### XCIII.—THE GREAT MASTERS OF THE PAST.

### Benvenuto Cellini.

"Cellini was one of the most extraordinary men, in an extraordinary age; his life written by himself is more amusing than any novel I know."

Thus speaks Horace Walpole, after studying Cellini's autobiography, and everyone who reads it must agree with him. Undoubtedly Benvenuto was a genius, an artist, and a wonderful mechanic as well; joined to these gifts, he was a braggart, an unscrupulous ruffian (by no means hesitating as to murder if it suited his ends), a dissembler, and despicable as to morality. There must, however, have been a soft corner in his heart, for to the last day of their needs he gave hospitality to his mother and his many sisters.

It is so difficult to judge leniently, and at the same time correctly, at this distance of time, the character and career of one who lived in the stormy period of the Renaissance.

### Was He so Untruthful as Represented?

J. Addington Symonds in the introduction to his able translation of Cellini's life, expresses the opinion that he was not such a groundless boaster as has been supposed, and gives the reasons for his belief, too voluminous for insertion here; to my mind they seem conclusive. The great artificer could have no expectation that he was writing for posterity; had it been so, his egregious vanity would certainly have led him to gild a halo of romance and fancy round many of the sordid details he relates with such uncompromising minuteness.

### His Life in Florence and Rome.

All the more interesting events of his stormy life took place in these two cities. Born at Florence in 1500, at number 6 Via de San Lorenzo, he was first apprenticed to a goldsmith named Bandinello, the father of that sculptor with whom he afterwards had such rancorous and embittered quarrels.

His roving spirit led him to throw up this comparatively quiet life, and he ran away to Rome in 1519.

He cannot have been an altogether abandoned character, in spite of his own confessions, for he was honoured with the friendship of Leonardo de Vinci and Michel Angelo, to both of whom he looked up all his life with exceeding reverence.

### The Sack of Rome.

It must have been during his second visit to Rome that he witnessed the entrance of the Constable Bourbon and the fearful sack of that city. He lays claim to having been the slayer of the Duke, and his account from its very simplicity bears the impress of truth:

"The army of the Duke having already appeared before the walls of Rome, Alessandro del Bena requested I would go with him to oppose the enemy. I accordingly complied, and taking one of the stoutest youths with us, we were afterwards joined on our way by a young gentleman of the name of Cecchino della Casa We came up to the walls of Campo Santo and there descried the great army. . . . . There was a remarkably thick mist. . . . ."

Then he goes on to recount his shooting of the constable. His friend had evidently shown the white feather and wished to return home.

"I thereupon reproved him, saying, 'Since you have brought me hither, I am determined to perform some manly action,' and levelling my arquebuse where I saw the thickest crowd of the enemy, I discharged it with a deliberate aim at a person who seemed to be lifted above the rest; but the mist prevented me from distinguishing whether he was on horseback or on foot. . . . . Having accordingly fired twice for the enemy's once, I cautiously approached the walls and perceived that there was an extraordinary confusion among the assailants, occasioned by our having shot the Duke of Bourbon. He was, as I understood afterwards, that chief personage whom I saw raised above the rest."

Had he invented this incident, he would have invested it with more pomp. The touch concerning "firing twice to the enemy's once" is truly Cellinesque.

### His Connection with Francis I.

It was in 1537 that he was summoned to Paris to assist the King in the embellishment of Fontainbleau, and it was during this period that his goldsmith's and jeweller's work prospered. The King and the reigning favourite, Madame d'Etampes, were his patrons, and as well as working in precious metals for them, he made the fine statue of Jupiter, about which, and his quarrel with the lady he gives us some not very refined details.

### His Imprisonment in San Angelo.

This occurred in 1538. He fell under a charge of embezzling some Papal jewels—it was undeniably a false accusation. He was certainly most immoral, but there is no shadow of credibility in this scandal. He effected his escape with a broken leg, and his account of the affair is as usual most dramatic. It was about this time that he gives a perfectly shameless account of his own brutality, and unexampled cruelty and scorn, to a wretched model of bad character, whom he had employed, which conduct again brought him into collision with the authorities. He married late in life, simply to provide himself with an obedient and attentive nurse; but he considers it such an unimportant affair that he does not even mention the circumstance, though we hear of it indirectly.

### The Casting of the Perseus.

We now approach the most interesting incident in Cellini's career. His great longing was to excel as a sculptor, and the Grand Duke Cosimo commissioned him to execute a group of Perseus with the head of Medusa. For this work he established himself in the Via del Rosaio in Florence. He chose this house, as he explains, because there was a large garden suitable for the erection of his furnace. Whilst engaged there on the work Francis I. wrote to ask for his services again, but Cellini refused, thus "I sat down to write an answer and filled nine pages of ordinary paper." I wish I could give his full account of the difficulties experienced in casting the Perseus. "No sooner was the furnace ready than I went to work with all diligence upon the casting of Medusa—that is the woman twisted in a heap at the feet of Perseus." Then he tells of making a funnel-shaped furnace and sinking the Perseus first in a pit, then banking it up with tons of wood filling the furnace with copper, pewter, etc., and setting it going. At the critical moment his workshop caught fire, whilst almost simultaneously a violent storm came on which cooled the furnace. His despair brought on a fever for which he was compelled to take to his bed, but he quickly rose again, kicked and cuffed every one all round, and rushed out to find the metal all curdling; he then flung every pewter and copper utensil in the house into the furnace, and with diligent stoking the mass again began to liquefy. His spirits then rose, and he straightway ate a large plateful of salad with hearty appetite.

All the world knows of his splendid success, and flocks to gaze upon the Perseus as it stands in the Loggia dei Lanzi at Florence, but comparatively few have read his charming account of the difficulties that beset him in carrying out this stupendous work.

He died at Florence in 1570, and, on the whole, Florence seems more to claim him as a personality than Rome.

